# PbSe Quantum Dot Doped Mode-Locked Fiber Laser

**DOI:** 10.3390/ma15217495

**Published:** 2022-10-26

**Authors:** Kaihua Wei, Libin Zhang, Hairong Zhu, Jia Hou, Zhousu Xu, Zhonghua Yu

**Affiliations:** 1 State Key Laboratory of Fluid Power and Mechatronic Systems, School of Mechanical Engineering, Zhejiang University, Hangzhou 310058, China; 2 Hangzhou Electric Connector Factory, Hangzhou 310052, China; 3 School of Automation, Hangzhou Dianzi University, Hangzhou 310018, China; 4 Institute of Intelligent Optoelectronic Technology, Zhejiang University of Technology, Hangzhou 310014, China

**Keywords:** quantum dot, wavelength tuning, laser, picosecond pulse, PbSe

## Abstract

Herein, a PbSe quantum dot-doped-mode-locked fiber laser is experimentally demonstrated. A PbSe quantum dot-doped fiber is prepared using a melting method and induced as a gain medium in our mode-locked fiber laser. By increasing the pump power, a stable pulse train is obtained with a pulse duration of 36 ps, a pulse repetition rate of 4.5 MHz, an average laser power of 9.8 mW, and a central wavelength of 1214.5 nm. The pulse duration can be changed by adjusting the PC or increasing the pump power. The maximum laser power obtained was 42.7 mW under the pump power of 800 mW. Our results prove that a quantum dot-doped-mode-locked fiber laser is achievable, which provides a new scheme to solve wavelength problem of rare-earth-doped mode-locked fiber lasers.

## 1. Introduction

Mode-locked fiber lasers—which offer ultra-short pulses, excellent beam quality, convenient thermal management, and high optical–optical conversion efficiency—are widely applied in laser microprocessing, optical nonlinear generation, and laser-based biomedicine [1,2,3,4,5]. To date, the use of rare-earth (RE) doping fibers is the conventional scheme to construct a mode-locked fiber laser; usually, a Yb-doped fiber is used to emit a wavelength of around 1 μm, an Er-doped fiber is used to generate a wavelength of about 1.5 μm, and a Tm-doped fiber is used to emit approximately 2 μm wavelengths [6,7,8].

However, the emission wavelength from REs has difficulty covering near-infrared and mid-infrared wavelengths seamlessly, which limits the application area of mode-locked fiber lasers. For instance, it has already been proven that a 1.7 μm wavelength is useful to improve the penetration depth in tissues [9,10]. Unfortunately, no mode-locked fiber laser is capable of producing this wavelength owing to the limitations of RE materials. As a result, a nonlinear generation technique is the only method for attaining a 1.7 μm ultra-short pulse [11,12]. In other words, there is a pressing need to seek a new gain medium material for emitting various wavelengths.

Quantum dots (QDs) are quasi-zero-dimensional semiconductor nanocrystal materials that can radiate any optical wavelength conveniently by changing their size [13,14,15]. In addition, QDs have large cross-sections, which implies high optical gain. Further, QDs can be easily embedded into a fiber [16,17]. Theoretically, QDs are an excellent gain medium for use in mode-locked fiber lasers.

In fact, a QD-doped fiber laser with continuous-wave (CW) emission has already been realized. In 2013, Cheng et al. reported a CW-operated-QD-doped fiber laser with a laser power of 6.36 mW under a 68 mW pump [18]. Alternatively, a QD-doped glass fiber has also been developed as the gain medium of an efficient fiber amplifier [16,19,20], which further promotes the development of QD-doped fiber amplifiers and lasers.

Among QDs, lead selenide (PbSe) QDs with a large exciton Bohr radius, high quantum yield, and a large absorption–emission cross-section are the ideal laser gain media. In this paper, a PbSe QD-doped-mode-locked fiber laser is experimentally demonstrated. A gain medium of the PbSe QD-doped fiber is prepared using a melting method and a two-step thermal process. The pulse is initiated via a quickly saturable absorber. An all-fiberized-ring-structured cavity is employed to build our PbSe QD-doped-mode-locked fiber laser. By increasing the pump power, a stable picosecond pulse train is obtained with a pulse duration of 36 ps, an average laser power of 9.8 mW, and a smooth spectrum is observed with a center wavelength of 1214.5 nm.

## 2. Materials and Methods

Our cavity uses PbSe QD-doped fiber as gain medium. The gain fiber is prepared using melting method and two-step thermal process. The materials of SiO_2_, B_2_O_3_, Al_2_O_3_, ZnO, AlF_3_, Na_2_O, PbO, and ZnSe are mixed and lapped in a crucible. PbO and ZnSe are the precursors of PbSe, while ZnO is employed to reduce the volatilization of Se. Afterwards, the crucible is put into a muffle furnace, in which it is held at 1400 °C for 1 h. A glass fiber is drawn from the melting material. Finally, a two-step heat treatment is applied to obtain PbSe QDs as well as control PbSe QDs sizes from the glass fiber in a tube furnace.

**Figure 1:**
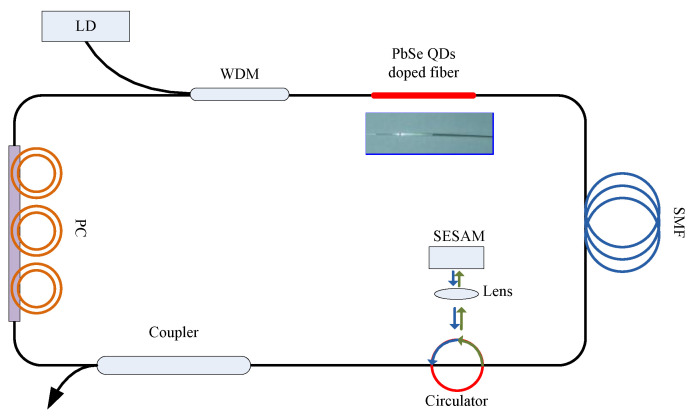
Configuration of PbSe QD-doped-mode-locked fiber laser.

Configuration of our mode-locked fiber laser is illustrated in Figure 1. Our resonator is based on a ring all-fiber configuration. A 2 cm long PbSe QD-doped fiber is pumped by a fiber-pigtailed 976 nm laser diode (LD) to generate and amplify noise-like pulses. A lens-coupled semiconductor-saturable absorber mirror (SESAM) is employed to narrow the pulse duration, while the traditional single-mode fibers are applied to connect components in our resonator as well as balance the dispersion and self-phase modulation (SPM). A polarization controller (PC) is applied to initial mode-locking process. The mode-locked pulses are emitted from a 10/90 fiber coupler. The total cavity length is approximately 17 m.

In our experiment, the absorption and fluorescence spectra are detected by a spectrophotometer (from Shimadzu, Kyoto, Japan) and a fluorescence spectrometer (from Edinburgh Instruments, Livingston, UK), respectively. The laser power, spectrum, and pulse are measured using an optical power meter (from Thorlabs, Sterling, Virginia) with a measurable power range from 10 μW to 1 W, an optical spectrum analyzer (OSA, from ANDO, Tokyo, Japan) with a spectral resolution of 0.05 nm, and an oscilloscope (from Tektronics, Tokyo, Japan) with a bandwidth of 12.5 GHz.

## 3. Results

**Figure 2:**
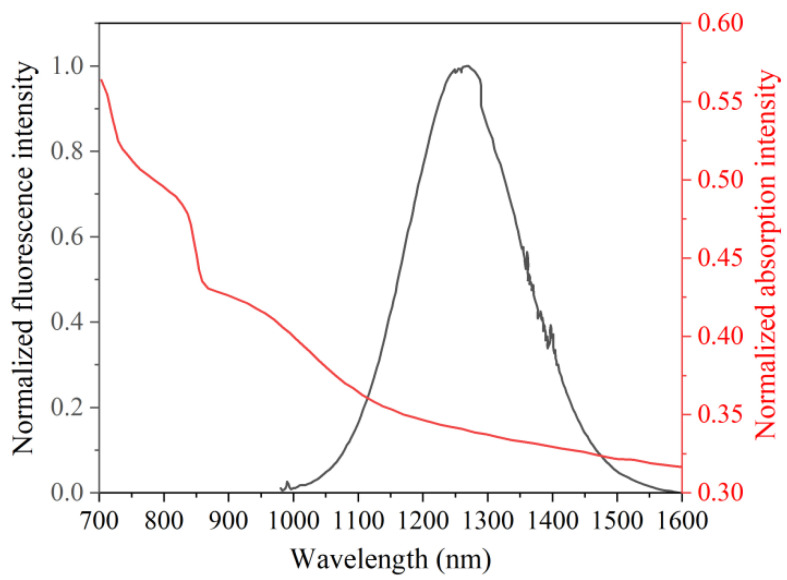
Absorption and fluorescence spectra from PbSe QD-doped glass fiber.

The absorption and fluorescence spectra are demonstrated in Figure 2. The absorption peak wavelength is around 950 nm, while that of the fluorescence spectrum is approximately 1250 nm. The Gauss-like fluorescence spectrum spans from 1000 nm to 1600 nm.

**Figure 3:**
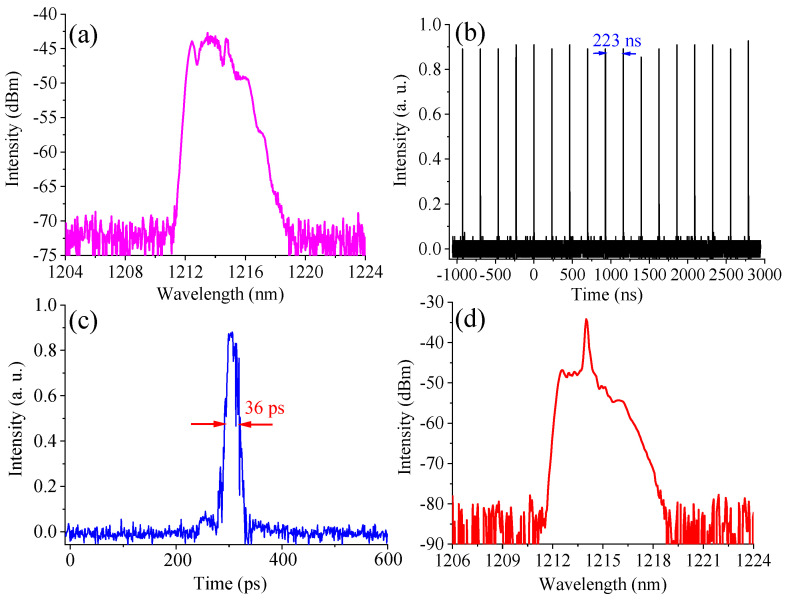
(**a**) Spectrum under pump power of 65 mW; (**b**) pulse train; (**c**) single pulse; (**d**) spectrum under pump power of 200 mW.

By increasing the pump power, an amplified spontaneous emission (ASE)-like spectrum was observed on the OSA at a pump power of 65 mW, as shown in Figure 3a; a noise-like signal is simultaneously illustrated on our oscilloscope. As the pump power is raised to 130 mW, an ambiguous pulse train is observed; however, this situation is unstable due to the inconsistent pulse duration and intensity between pulses as well as the harsh pulse curve. Under a pump power of 200 mW, a stable mode-locked pulse train was demonstrated with a pulse repetition rate of around 4.5 MHz, as demonstrated in Figure 3b. Figure 3c illustrates a single pulse with a pulse duration of 36 ps. Meanwhile, an irregularly shaped spectrum was also obtained with a sharp peak, which has a center wavelength of 1214.5 nm and a spectral full width at half maximum (FWHM) of 0.25 nm.

**Figure 4:**
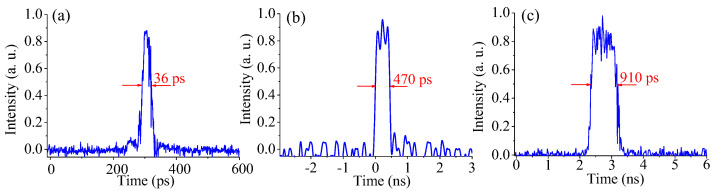
Various mode-locking statuses with respect to different PC angle of (**a**) 13 degrees, (**b**) 28 degrees and (**c**) 41 degrees.

By tuning the PC, various mode-locking statuses are obtained, as shown in Figure 4. Under the same pump power of 200 mW, the pulse characteristics can be changed by solely tuning the PC. The pulse durations of 36 ps, 470 ps, and 910 ps were obtained corresponding to the average laser power of 9.8 mW, 10.3 mW, and 11 mW, respectively.

**Figure 5:**
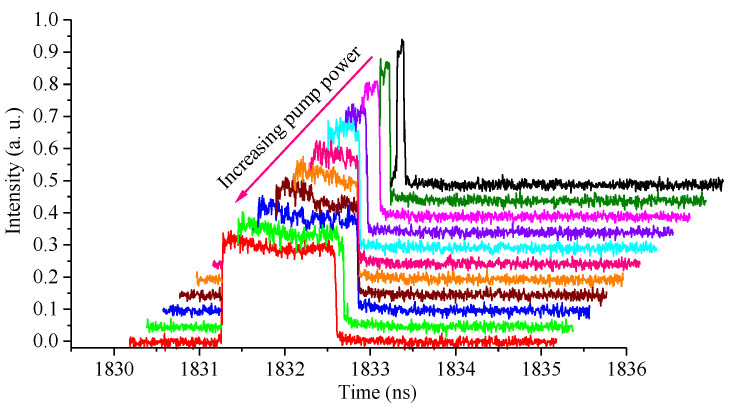
Changes of pulse characteristics by increasing pump power.

The pulse duration can also be changed by increasing the pump power. As the PC is fixed and the pump power is raised from 200 mW to 800 mW, the pulse duration is increased from 36 ps to 1.2 ns, as shown in Figure 5. Square pulses are observed over a pump power of 350 mW.

**Figure 6:**
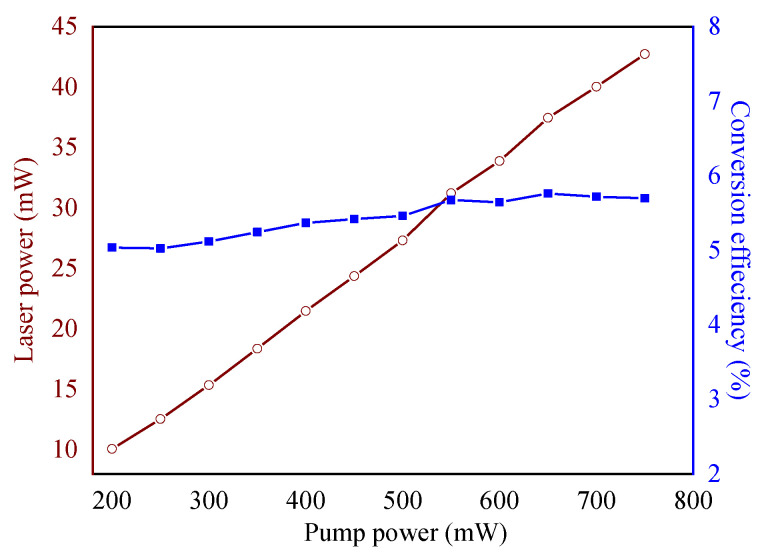
Average laser power and conversion efficiency with respect to pump power.

The average laser power and conversion efficiency as a function of pump power is shown in Figure 6. It is evident that the laser power is linearly raised by increasing the pump power, while the optical–optical conversion efficiency is nonlinearly raised. The maximum laser power is obtained as 42.7 mW under a pump power of 800 mW. The highest conversion efficiency is 5.72%, corresponding to the pump power of 700 mW.

## 4. Discussion

In summary, a PbSe QD-doped-mode-locked fiber laser has been experimentally built and realized. Thanks to the large emission cross-section of the PbSe QDs allowing for the giant round-trip gain, a short PbSe QD-doped fiber of 2 cm was applied in our resonator. A stable pulse train was obtained with a pulse duration of 36 ps and a pulse repetition rate of 4.5 MHz. The pulse duration can be changed by adjusting the PC or increasing the pump power. The maximum laser power obtained was 42.7 mW under a pump power of 800 mW. Our results indicate that PbSe QDs constitute an efficient gain medium in mode-locked fiber lasers. Due to quantum size effects in the PbSe QDs, our new resonator scheme is promising for solving wavelength dilemmas in RE-doped-mode-locked fiber lasers.

## Data Availability

Not applicable.
